# Atypical delayed auditory feedback effect and Lombard effect on speech production in high-functioning adults with autism spectrum disorder

**DOI:** 10.3389/fnhum.2015.00510

**Published:** 2015-09-22

**Authors:** I-Fan Lin, Takemi Mochida, Kosuke Asada, Satsuki Ayaya, Shin-Ichiro Kumagaya, Masaharu Kato

**Affiliations:** ^1^Human and Information Science Laboratory, NTT Communication Science LaboratoriesAtsugi, Japan; ^2^Research Center for Advanced Science and Technology, The University of TokyoTokyo, Japan; ^3^Center for Baby Science, Doshisha UniversityKizugawa, Japan

**Keywords:** autism, delayed auditory feedback, lombard effect, audio-motor coordination, feedback control, feedforward control, speech production

## Abstract

Individuals with autism spectrum disorder (ASD) show impaired social interaction and communication, which may be related to their difficulties in speech production. To investigate the mechanisms of atypical speech production in this population, we examined feedback control by delaying the auditory feedback of their own speech, which degraded speech fluency. We also examined feedforward control by adding loud pink noise to the auditory feedback, which led to increased vocal effort in producing speech. The results of Japanese speakers show that, compared with neurotypical (NT) individuals, high-functioning adults with ASD (including Asperger’s disorder, autistic disorder, and pervasive developmental disorder not otherwise specified) were more affected by delayed auditory feedback but less affected by external noise. These findings indicate that, in contrast to NT individuals, those with ASD relied more on feedback control than on feedforward control in speech production, which is consistent with the hypothesis that this population exhibits attenuated Bayesian priors.

## Introduction

Autism spectrum disorder (ASD) has been defined in DSM-5 as a group of conditions in which the core symptoms are: (1) impairments in social interaction and communication and (2) restricted and repetitive behaviors. Although there is heterogeneity in the speech production of individuals with ASD, a certain percentage exhibit more speech errors in their childhood than neurotypical (NT) individuals, and some of these speech errors do not improve with age (Cleland et al., 2010; Oller et al., 2010; Shriberg et al., 2011). Studies show that these speech errors observed in individuals with ASD are not caused by oral motor dysfunction or dysfunctional speech planning/programming, such as spatiotemporal vowel errors, uncommon phoneme distortions, or slow speech rate (Shriberg et al., 2011). On the other hand, based on the consistent findings of large variation in the loudness/pitch of their speech (Shriberg et al., 2011), we hypothesized that their speech production might rely heavily on auditory feedback, so tiny variations in the loudness/pitch of their speech due to unexpected distortion might be amplified by overshooting adjustments.

Speech production is a complicated process, which involves the motor system, the auditory system, the somatosensory system, and their coordination. Speech production relies on feedback control and feedforward control (Hickok, 2012). Feedback control relies on monitored somatosensory and auditory feedback. If there is a mismatch between perceived and predicted sensory consequences, the error-correction mechanism changes the motor commands based on the mismatch. For example, when the auditory feedback of our own voice is artificially pitch-shifted, the auditory-motor system compensates for these perturbations by modulating the motor commands, which results in the pitch-shifting of our own voice (Burnett et al., 1998; Chang et al., 2013), and the compensation of the auditory-motor system for delayed auditory feedback (DAF) results in increased phonation time and phonation errors (Yates, 1963). On the other hand, feedforward control relies on the previously learned correlation between motor commands and their outcomes (i.e., sensory-motor neural mappings). Because feedforward control allows the motor system to make plans for future movements, it is crucial for fluent speech. For example, it is hypothesized that stuttering involves excessive reliance on auditory feedback control due to poor feedforward control (Civier et al., 2010). Feedforward control is also important for adaptation in an adverse environment because it evaluates the disturbances beforehand and changes the motor plan to deal with the environmental changes. For example, when there is loud background noise, we would observe increased intensity and phonation duration in speaker’s speech production (Junqua, 1996; Zollinger and Brumm, 2011). The modulation of speech production in a noisy environment, known as the Lombard effect, was discovered in 1909 by an otolaryngologist, Etienne Lombard. The Lombard effect is suggested to make communication in a noisy environment effective (Zollinger and Brumm, 2011).

Since the deficits in social interaction and communication observed in individuals with ASD might be partially explained by an increased number of speech errors, it is important to investigate the mechanisms of these speech errors. Here we investigated auditory-motor coordination in speech production in Japanese-speaking individuals with ASD. Specifically, we examined the way in which they rely on feedback control by observing how they react to DAF and the way in which they rely on feedforward control by observing how they react to increased background noise. Some researchers argue that individuals with ASD have attenuated Bayesian priors, so they tend to perceive the world more accurately than NT individuals, while perception in NT individuals is modulated by prior experience (Pellicano and Burr, 2012). In other words, compared to NT individuals, individuals with ASD might put less weight on the information gained from their prior experience. Here we extended this argument from perception to sensory-motor interaction: If individuals with ASD put less weight on their prior information, they might rely less on feedforward control and more on feedback control.

Previous visuo-motor studies have suggested that individuals with ASD rely more on feedback control than on feedforward control (Schmitz et al., 2003; Martel et al., 2014). In addition, a previous study using pitch-shifted auditory feedback also found that a subgroup of individuals with ASD have larger responses to perturbed auditory feedback (Russo et al., 2008), which indicates that some individuals with ASD rely more on feedback control than on feedforward control in speech production. Another previous study found no difference between the ASD group and the control group for DAF and the Lombard effect (Nober and Simmons, 1981), but they only tested a small number of participants. Here we tested a larger number of participants. In addition to the voiced DAF experiment, we asked the participants to produce whispered speech in a separate DAF experiment. Therefore, we could examine the effect of speaking modes on responses to DAF. Moreover, because the corollary discharges from the motor controller influence how the motor system calculates the errors in movement and influence how we perceive the agent giving the motor commands (i.e., self-agency attribution), we collected the participants’ evaluation of the self-agency attribution of DAF to examine the influence of corollary discharges.

## Materials and Methods

### Participants

Fourteen participants with ASD and 15 NT participants joined this study, but three participants with ASD did not finish the experiment because they could not tolerate the background noise used in the experiments, one NT participant was excluded because of severe hearing loss (defined as pure-tone hearing thresholds above 40 dB HL at more than one audiometric frequency), and another NT participant was excluded because his autistic traits (AQ, measured by Autism-Spectrum Quotient Questionnaire) were too high (his AQ score = 34, and the cut-off point of AQ for ASD is 33 for the Japanese version of Autism-Spectrum Quotient Questionnaire). Finally, the results from 11 participants with ASD and 13 NT participants were included in the analysis of the voiced DAF experiment, the Lombard effect experiment, and subjective report for DAF. For the whispered DAF experiment, two NT participants had extreme difficulty in producing whispered speech when loud background noise was present (i.e., they could only produce voiced speech under noise), so only results from 11 participants with ASD and 11 NT participants were included in the analysis of the whispered DAF experiment.

These 11 participants with ASD and 13 NT participants were matched by age and intelligence quotient (IQ). Verbal IQ (VIQ) and performance IQ (PIQ) were measured by WAIS-III or WAIS-R (Wechsler, 1981; Shinagawa et al., 1990; Fujita et al., 2006). The Autism Diagnostic Observation Schedule (ADOS) was used to evaluate the severity of symptoms in participants with ASD. The cut-off point of ADOS for ASD is 7, and only half of our participants with ASD had scores above the cut-off point despite that all of them were diagnosed as ASD by medical doctors. The distribution of ADOS scores in the ASD group (*N* = 10 because one ASD participant was not evaluated for ADOS) did not show significant difference from the cut-off point of ADOS [two-tailed *t*-test showed that *t*(9) = 1.07, *p* = 0.31]. We split the ASD group into two groups based on the cut-off point in ADOS, but there was no significant between-group difference in their responses in the following experiments. As a result, we still put all their results together in the analyses described in the Results section. **Table 1** provides detailed information about the participants. The participants with ASD were recruited through a self-help group for people with developmental disabilities, and their ASD diagnosis was provided by hospitals based on DSM-IV-TR (APA, 2000) (four were diagnosed with Asperger’s disorder, one was diagnosed with autistic disorder, and six were diagnosed with pervasive developmental disorder not otherwise specified).

**Table 1:** Mean-group matching data for the autism spectrum disorder (ASD) and neurotypical (NT) participants.

Mean ± Standard Deviation (range)	ASD	NT	*t*-test
Gender (female : male)	9:2	8:5	–
Age	40.45 ± 11.47 (18–54)	39.15 ± 9.77 (20–52)	*p* = 0.38
Full intelligence quotient (IQ)	111.09 ± 12.41 (91–124)	107.38 ± 12.16 (90–128)	*p* = 0.49
Performance IQ (PIQ)	103.82 ± 17.02 (71–126)	102.54 ± 12.89 (80–122)	*p* = 0.78
Verbal IQ (VIQ)	115.18 ± 14.78 (92–136)	110.08 ± 12.24 (96–137)	*p* = 0.44
Autism-Spectrum Quotient Questionnaire (AQ; Baron-Cohen et al., 2001)	34.8 ± 9.21^†^ (19–43)	17.69 ± 5.3 (9–27)	*p* = 0.0001
Autism Diagnostic Observation Schedule (ADOS; Lord et al., 2000)	8.8 ± 5.31^†^ (2–16)	–	–

*^†^Lacked the data from one participant*.

Written informed consent was obtained from all participants before we conducted the experiments. All procedures were conducted in accordance with the Declaration of Helsinki and approved by the ethics committee of NTT Communication Science Laboratories. All of the participants were naive to the purposes of the study. The participants were paid for their time. The participants all had normal hearing, defined as pure-tone hearing thresholds of 20 dB HL or less at audiometric frequencies between 1 and 4 kHz, except for one female participant with ASD (her hearing levels were 30 dB for one ear at 1 kHz and for both ears at 2 kHz). The data from this participant were still included because her hearing loss was mild and the exclusion of her data did not change the results of the statistical analysis (see Supplemental Materials).

### Voiced DAF Experiment

On each trial, participants were instructed to read ‘ma-mi-mu-me-mo’ naturally and without pausing into a microphone in less than 5 s. In each session, there were five delay conditions (delays of 0.05, 50, 100, 200, and 400 ms), and five trials for each delay condition, giving a total of 25 trials. The order of these delay conditions was randomized in each session. In total there were four sessions.

To mask their original auditory feedback, 68.8-dB SPL pink noise was sent to their headphones continuously. The recorded speech was delayed and amplified by 10 dB before being sent to the headphones. The phonemes of the recorded speech signals were labeled individually in Praat based on the spectrogram and heard sounds (Boersma and Weenink, 2014) by two technicians who did not know the purpose of the experiment and the details of the recorded materials. The phoneme contents and their start and end points were labeled, and the labeled data were then processed in MATLAB (R2010b; The MathWorks Inc.) to compute the syllable number (by combining two phonemes) and phonation duration. There should be five syllables (‘ma,’ ‘mi,’ ‘mu,’ ‘me,’ ‘mo’) if the participants made no mistakes.

### Whispered DAF Experiment

After the voiced DAF experiment, participants took a short break. Before the whispered DAF experiment started, participants practiced how to whisper with or without noise delivered by the headphones. They were told that there should be no vocal fold vibrations when they whispered. They then practiced voiced speech and whispered speech while touching their necks to feel for vocal fold vibrations.

The whispered DAF experiment had the same experiment set-up and procedures as the voiced DAF experiment except that participants were instructed to whisper ‘ma-mi-mu-me-mo’ into the microphone. To mask their original auditory feedback, 58.5-dB SPL pink noise was sent to their headphones continuously. Because it was extremely difficult to segregate the phonemes in the whispered speech signals, after the start and end points of the speech signals in each trial were labeled in Praat, the phoneme contents were labeled as a group instead of being labeled individually. The labeled data were then processed in MATLAB as the data collected in the voiced DAF experiment.

### Lombard Effect Experiment

In this experiment, the participants were instructed to read out the number presented on the computer screen as soon as possible. In each session, there were 14 numbers (randomly chosen from 2, 3, and 4, which are pronounced ‘ni,’ ‘san,’ and ‘yon’ in Japanese) presented on the computer screen one after another, one second apart. The participants finished four sessions under the quiet condition and four sessions under the noise condition, and the order of the quiet and noise sessions was randomized. In the noise sessions, 69-dB SPL pink noise was sent continuously to the headphones. The recorded speech signals were analyzed with MATLAB: the speech signals were segregated from the silence periods using the root mean square amplitude of the waveforms as the criterion, and then their individual duration and sound pressure level were computed.

### Subjective Report for DAF

For each delay condition (voiced with five different delays, in a randomized order), the participants read a randomly chosen short sentence and evaluated three self agency-related descriptions using a 7-point scale (from strongly disagree to strongly agree): It seemed as though the sound I heard was (1) made by me (2) made by someone else (3) made by me but altered.

### Apparatus

All auditory experiments were conducted in a sound-insulated booth. These experiments were controlled by a computer. The participants’ speech was collected with a microphone (Rode NT2-A). A mixer (Soundcraft EPM6) was used to send the collected speech to a noise filter (Behringer Multigate Pro XR4400), and the filtered speech was mixed with pink noise (generated by an ST-NG1 white and pink noise generator). The filtered speech signals were sent to an audio interface (Roland EDIROL) and a computer for recording. In the DAF experiment, the filtered speech signals were also sent to a TDT RP2.1 and a TDT PA5 (Tucker-Davis Technologies Inc.) to adjust the delay before being mixed with pink noise. The processed speech signals (with delay or not) were then sent to the participants through headphones (Sennheiser HDA200) that had a passive attenuation (for environmental sounds) of more than 20 dB in the spectral region above 500 Hz.

## Results

The ANOVA analyses were conducted with SPSS v.19 (IBM, USA), and other statistical analyses were conducted with MATLAB. All the conducted *t*-tests were two-tailed tests.

### Voiced DAF Experiment

When there was no perceived delay (i.e., for the 0.05-ms delay condition), the phonation duration was significantly longer in the ASD group (mean = 0.99, 95% CI = [0.87, 1.1]) than in the control group (mean = 0.85, 95% CI = [0.78, 0.92]) [*t*(22) = 2.29, *p* = 0.03]. On the other hand, when there was no perceived delay, there was no significant between-group difference in syllable number [*t*(22) = 0.92, *p* = 0.37]. These findings indicate that when there was no perceived delay, although individuals with ASD had longer speech production, they did not make more syllable number errors. Since the phonation duration for a 0.05-ms delay was significantly longer in the ASD group than in the control group, the duration ratio (the phonation duration with a certain delay divided by the phonation duration with a 0.05-ms delay) was used instead of the raw duration data in further analysis.

**FIGURE 1:**
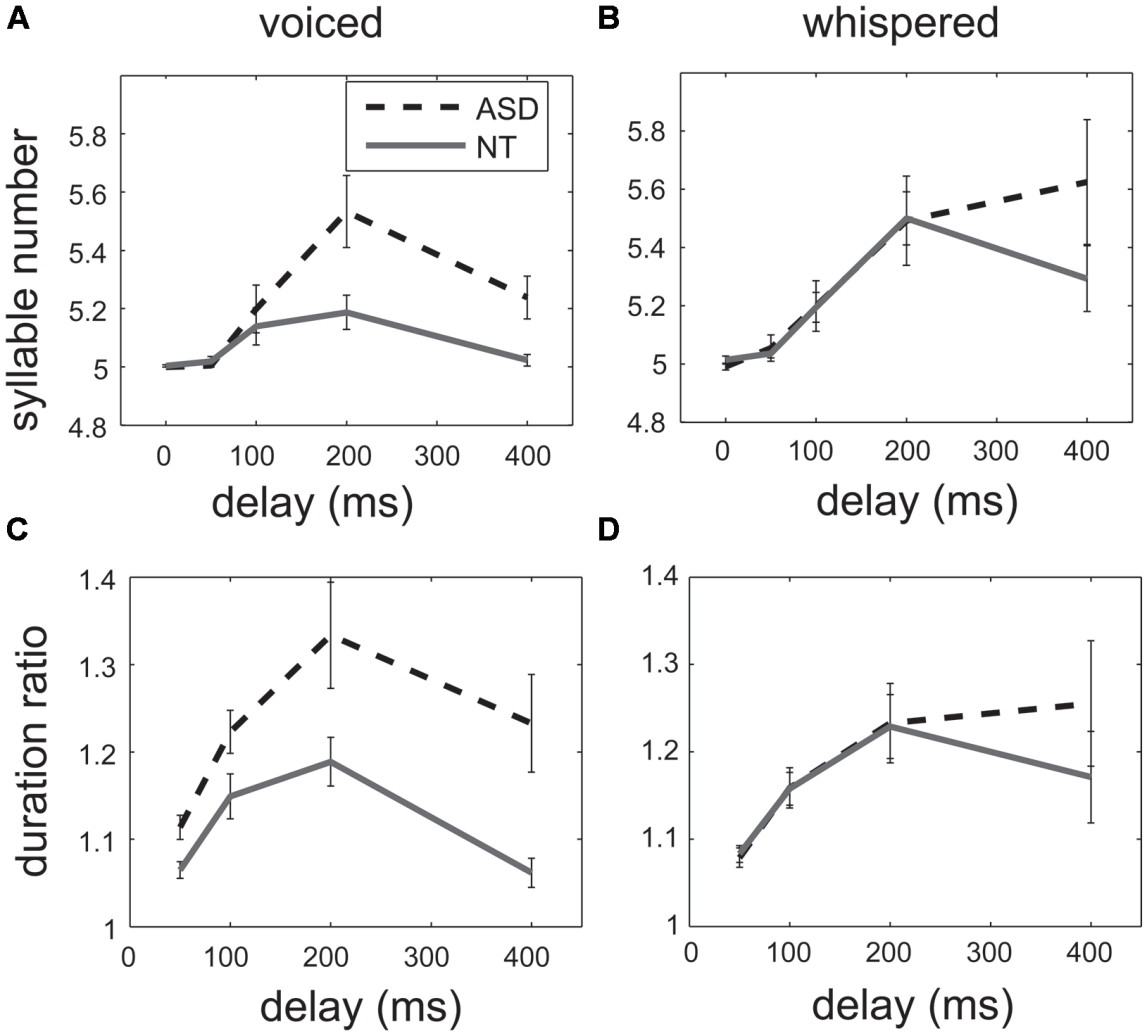
**The effect of the delay of auditory feedback on syllable number (above) and duration ratio (below) in two groups in the voiced delayed auditory feedback (DAF) experiment **(A,C)** and in the whispered DAF experiment (B,D).** While the delay increased up to 200 ms, the syllable number and the duration ratio increased in both groups, indicated as mean ± standard error. For the voiced DAF experiment, the syllable number and the duration ratio increased more in the ASD group (black dashed lines) than in the control group (gray solid lines), but there was no such difference for the whispered DAF experiment.

**Figures 1A,C** shows that the syllable number and the duration ratio increased in both groups and peaked at 200-ms delay. In addition, the responses to DAF were larger in the ASD group (the black dash lines) than in the control group (the gray solid lines).

To investigate the effect of the group and delay in different speech production modes, two mixed-design ANOVAs were conducted with factors Group and Delay for the syllable number and the duration ratio. In the ANOVA analysis, the between-subject factor Group had two levels: the ASD group and the control group; the within-subject factor Delay, which refers to the different delays used in the DAF experiments, had either four levels for the analysis of duration ratio or five levels for the analysis of syllable number. Delay was a significant factor [*F*(2.44,53.62) = 16.31, *p* < 0.001 for the syllable number, *F*(1.99,43.66) = 21.84, *p* < 0.001 for the duration ratio], and Group was also a significant factor [*F*(1,22) = 6.03, *p* = 0.02 for the syllable number, and *F*(1,22) = 8.44, *p* = 0.01 for the duration ratio]. Across different delays, the syllable number was larger in the ASD group (mean = 5.2, 95% CI = [5.12, 5.27]) than in the control group (mean = 5.08, 95% CI = [5.01, 5.14]), and the duration ratio was larger in the ASD group (mean = 1.23, 95% CI = [1.17, 1.28]) than in the control group (mean = 1.12, 95% CI = [1.06, 1.17]). Similarly, a significant interaction between Delay and Group was observed [*F*(2.44,53.62) = 4.67, *p* = 0.01 for the syllable number, and *F*(1.99,43.66) = 3.5, *p* = 0.04 for the duration ratio]. The ANOVA results support that the responses to DAF were larger in the ASD group than in the control group.

To investigate the influence of bone conduction and air conduction for auditory feedback, the pairwise linear correlation between the speech sound pressure level and the effect of DAF was calculated. The recorded peak sound pressure level (59–85 dB SPL) was found to be correlated with the syllable number and duration ratio at 200-ms delay in the ASD group (syllable number: *r* = 0.68, *p* = 0.02; duration ratio: *r* = 0.74, *p* = 0.01) but not in the control group (syllable number: *r* = 0.19, *p* = 0.54; duration ratio: *r* = –0.29, *p* = 0.33).

### Whispered DAF Experiment

For the 0.05-ms delay condition, there was no significant difference in phonation duration [*t*(20) = 0.75, *p* = 0.46] or in syllable number [*t*(20) = 1.28, *p* = 0.21]. However, to make the data analysis comparable between the voiced DAF experiment and whispered DAF experiment, the duration ratio was still used in the following analysis.

Similar to the voiced DAF experiment, **Figures 1B,D** shows the syllable number and the duration ratio increased in both group and peaked at 200-ms delay, but there was not much difference between responses to DAF in the ASD group (the black dash lines) and in the control group (the gray solid lines).

To investigate the effect of the group and delay in different speech production modes, two similar ANOVAs were conducted with factors Group and Delay for the syllable number and the duration ratio for the voiced and whispered experiments. Delay was a significant factor [*F*(1.45,28.91) = 13.27, *p* < 0.001 for the syllable number, and *F*(1.19,23.88) = 12.88, *p* = 0.001 for the duration ratio], but Group was not a significant factor [*F*(1,20) = 0.52, *p* = 0.48 for the syllable number, and *F*(1,20) = 0.22, *p* = 0.64 for the duration ratio]. The interaction between Delay and Group was not significant [*F*(1.45,28.91) = 1.43, *p* = 0.25 for the syllable number, and *F*(1.19,23.88) = 1.26, *p* = 0.28 for the duration ratio]. Furthermore, the recorded peak sound pressure level (42–60 dB SPL) was not correlated with the syllable number or the duration ratio at 200-ms delay.

### Lombard Effect Experiment

**FIGURE 2:**
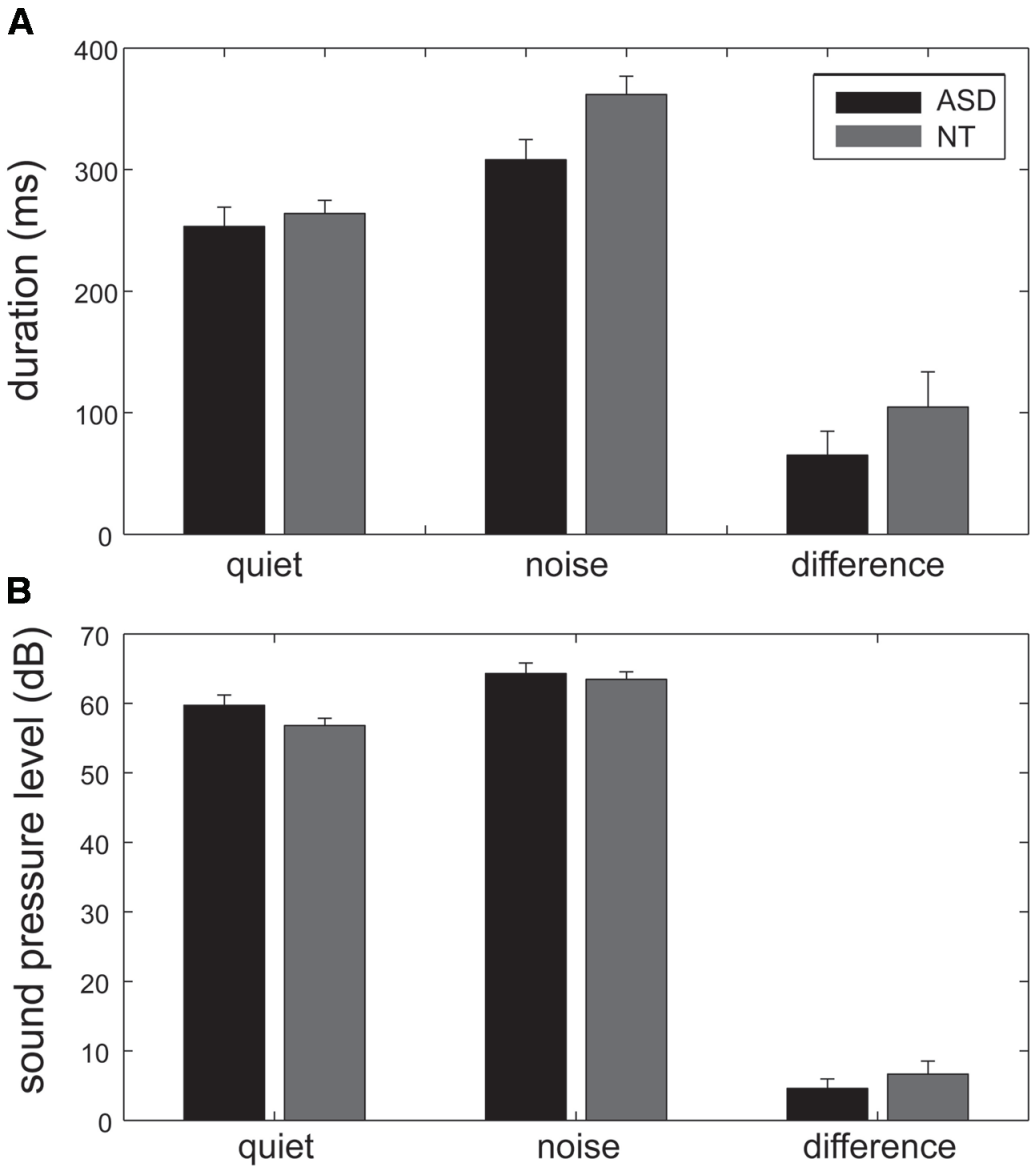
**The phonation duration and the sound pressure level measured for the quiet and noisy conditions in the two groups**. When the participants’ own speech was sent back to them with background noise, both their phonation duration **(A)** and sound pressure level **(B)** increased, indicated as mean ± standard error. The Lombard effect, which was the increase in phonation duration and sound pressure level for the noisy condition (the rightmost bars), was significantly smaller in the ASD group (black bars) than in the control group (gray bars).

As the result of some speech errors, there were only 13 instead of 14 spoken numbers in 2% of the sessions. The phonation duration and the sound pressure level (calculated as dB SPL) were used to evaluate the Lombard effect. For each participant, the phonation duration and the sound pressure level of the spoken words were averaged across repetitions and then averaged across different words (**Figure 2**).

To investigate the effect of the group and noise condition, two mixed-design ANOVAs were conducted with factors Group and Condition for the duration and the sound pressure level of their speech. For both phonation duration and sound pressure level, Condition was a significant factor [*F*(1,22) = 119.3 and 105.49, *p* < 0.001, respectively], and the interaction between Group and Condition was significant [*F*(1,22) = 100.9 and 89.26, *p* < 0.001, respectively]. Group was a significant factor for the sound pressure level [*F*(1,22) = 5.59, *p* = 0.03] but not for the phonation duration [*F*(1,22) = 0.023, *p* = 0.64].

The Lombard effect is defined as the difference between the phonation duration/sound pressure levels in the quiet and noise conditions. The follow-up *t*-tests showed that when loud background noise was added to the auditory feedback, both the ASD group and the control group exhibited a significantly increased phonation duration (ASD group: mean of increased duration = 65 ms, 95% CI = [47 ms, 83 ms], *t*(10) = 7.8, *p* < 0.001; NT group: mean of increased duration = 105 ms, 95% CI = [84 ms, 125 ms], *t*(12) = 10.96, *p* < 0.001) and sound pressure level (ASD group: mean of increased sound pressure level = 4.58 dB, 95% CI = [2.79 dB, 6.37 dB], *t*(10) = 5.7, *p* < 0.001; NT group: mean of increased sound pressure level = 6.68 dB, 95% CI = [5.26 dB, 8.08 dB], *t*(12) = 10.31, *p* < 0.001).

Although both the ASD group and the control group exhibited a significant Lombard effect, the Lombard effect measured by phonation duration was significantly longer in the control group than in the ASD group [*t*(22) = 3.07, *p* = 0.01]. Although the between-group difference in the Lombard effect measured by the sound pressure level was borderline significant [*t*(22) = 2.05, *p* = 0.05], it became significant [*t*(20) = 3.19, *p* = 0.01] after the removal of the outliers (one ASD participant and one NT participant whose Lombard effect measured by sound pressure level was larger than the cross-subject average Lombard effect measured by sound pressure level plus two standard deviations in each group).

### Subjective Report for DAF

The evaluation for the three questions in all the participants showed consistent trends, namely that as the delay of auditory feedback increased, they felt that the auditory feedback was less like their own sound (Q1), and was more like other people’s sounds (Q2) and their own sound but altered (Q3).

Three two-way mixed-design ANOVAs, with factors Group and Delay, were conducted along with an evaluation of each description in the questionnaire. These ANOVAs revealed that Delay has a significant effect for all descriptions [*F*(4,88) = 5.69, 5.14, and 5.98, *p* < 0.001, *p* = 0.001, and *p* < 0.001 for Q1, Q2, and Q3, respectively], but the effect of Group [*F*(1,22) = 0.1, 0.15, and 0.45, *p* = 0.76, 0.7, and 0.45 for Q1, Q2, and Q3, respectively] and the interaction between Delay and Group [*F*(4,88) = 1.45, 1.77, and 0.19, *p* = 0.226, 0.14, and 0.94 for Q1, Q2, and Q3, respectively] were not significant.

## Discussion

This study investigated auditory-motor coordination in individuals with ASD by examining feedback control and feedforward control in their speech production. In the voiced DAF experiment, the auditory feedback was delayed to test feedback control, and the ASD group produced significantly more syllabic numbers and had longer phonation duration than the control group in the voiced DAF experiment. In the Lombard effect experiment, loud noise was mixed with the auditory feedback to test feedforward control, and the ASD group showed less change in speech duration and sound pressure level than the control group. These results support the hypothesis that speech production in individuals with ASD relies more on error corrective feedback control than on environment responsive feedforward control.

When considering the increased responses to DAF in the ASD group, we first investigated the possibility that individuals with ASD over-evaluated the mismatch between the perceived and predicted sensory consequences due to imprecise corollary discharges from the motor controller (Goldberg et al., 1997). Because the corollary discharges carry information about sensory consequences that can be compared with perceived sensory consequences and produce a sense of self-agency (Gallagher, 2000), we evaluated self-agency attribution for DAF. The subjective reports for DAF show that participants with ASD and NT participants had a similar evaluation of the sense of self-agency. Our findings are consistent with a previous study that found similar self-generated tickling perception in the ASD and control groups, which also rejects the imprecise corollary discharges in individuals with ASD (Blakemore et al., 2006).

The whispered DAF experiment was designed to investigate whether different speaking modes changed the effect of DAF. A previous study showed that bilingual speakers speak faster and stutter less under DAF when they are speaking a more rather than a less familiar language (MacKay, 1970). Similar to speaking the second language, whispering is a less familiar speaking mode. While ASD individuals relied on feedback control for both voicing and whispering modes, NT individuals might rely less on feedback control in the voicing mode because of familiarity and rely more on feedback control in the whispering mode because of unfamiliarity.

Although it is tempting to think that the between-group difference for the voiced DAF experiment and the disappearance of the between-group difference for the whispered DAF experiment were due to the absence of bone-conducted auditory feedback for the whispered DAF experiment, it might not be the case. First, the air- and bone-conducted auditory feedback for the voiced DAF experiment was assumed to be blocked and disrupted by the loud noise sent to the headphones with a closed-form design. Second, the finding that there was a significant positive correlation between the recorded sound pressure level and the effect of DAF with a 200-ms delay in the ASD group for the voiced DAF experiment showed that an increased sound pressure level did not help the participants with ASD avoid the disturbing effect caused by DAF. Instead, the participants who were severely influenced by DAF generated the loudest speech production.

The reduced Lombard effect observed in individuals with ASD is consistent with our hypothesis that, compared with the control group, their speech production relied less on feedforward control so their motor plan changed less in the presence of environmental noise. In other words, individuals with ASD might have more difficulty in adapting their motor output in a context-dependent way. Nevertheless, there is another plausible explanation: if the non-reflex component in the Lombard effect is absent or smaller in individuals with ASD due to the absence of theory of mind (Lombardo and Baron-Cohen, 2011), the overall Lombard effect would be smaller in the ASD group. Although the Lombard effect has a brainstem origin (Nonaka et al., 1997; Hage et al., 2006), the finding that neural activities in the auditory cortex correlate with the generation of the Lombard effect indicates cortical involvement (Eliades and Wang, 2012). Behavioral studies also show that the Lombard effect varies with speech content (Patel and Schell, 2008) and in different contexts (communicative or not) (Amazi and Garber, 1982; Garnier et al., 2010). Moreover, although it is difficult to suppress the Lombard effect intentionally, it is possible to suppress it with training (Pick et al., 1989). In short, the Lombard effect is not simply a reflex, and its amplitude is modified based on the estimated difficulty listeners face in understanding speech in a noisy environment. Therefore, if individuals with ASD are not able to estimate the clarity of their speech heard by others in a noisy environment, they will retain the reflex component of the Lombard effect but their overall Lombard effect will be smaller than that in the control group.

In summary, our observations suggest that speech production in individuals with ASD relies more on feedback control than on feedforward control. This finding is consistent with our extension of the argument that individuals with ASD put less weight on prior information (Pellicano and Burr, 2012). However, there is limitation for our interpretation of the results. Because many participants with ASD expressed their intolerance for loud noise, we did not use very loud background noise (such as 90 dB SPL) in the voiced DAF experiment. Therefore, while some participants had very loud speech production, it was difficult to control the air- and bone-conducted auditory feedback. Although we showed that loud speech production did not help reduce the responses to DAF in the ASD group, it could not be ruled out that these two groups of participants utilized air- and bone-conducted auditory feedback in a different way. In addition, future studies should investigate which parts of the brain areas responsible for speech production (Hashimoto and Sakai, 2003; Takaso et al., 2010; Hickok et al., 2011) are affected in ASD and verify our finding that speech production in individuals with ASD relies more on feedback control than on feedforward control on a neural basis (Parker Jones et al., 2011).

## Author Contributions

IL, TM, MK designed the experiments; IL, KA, SA, SK, and MK conducted the experiments and collected the data; IL analyzed the data and drafted the manuscript; All the authors revised the manuscript and approved the manuscript.

## Conflict of Interest Statement

The authors declare that the research was conducted in the absence of any commercial or financial relationships that could be construed as a potential conflict of interest.
