# Supplementary material for: Atypical delayed auditory feedback effect and Lombard effect on speech production in high-functioning adults with autism spectrum disorder
Source: Front Hum Neurosci. 2015 Sep 22;9:510. doi: 10.3389/fnhum.2015.00510 (PMC4585204; doi:10.3389/fnhum.2015.00510)
Supplement: Supplementary file 1 [file Presentation_1.PDF]

Supplementary analysis

The following analysis show the results while the data from the ASD participant with mild hearing loss was excluded.

Delayed auditory feedback experiment

When there was no perceived delay (i.e., for the 0.05-ms delay condition), the phonation duration was significantly longer in the ASD group than in the control group for the voiced DAF experiment ($t(21)=2.39$, $p=0.03$) but not for the whispered DAF experiment ($t(19)=0.79$, $p=0.44$). On the other hand, when there was no perceived delay, there was no significant between-group difference in syllable number (voiced DAF experiment: $t(21)=0.87$, $p=0.39$; whispered DAF experiment: $t(19)=1.27$, $p=0.22$).

Four ANOVAs were conducted with factors Group and Delay for the syllable number and duration ratio for the voiced DAF experiment and whispered DAF experiment (Summarized in Table S1).

Table 2 The results of ANOVAs conducted for the results for the voiced DAF experiment and whispered DAF experiment.

		Voiced DAF experiment		Whispered DAF experiment	
		Syllable number	Duration	Syllable number	Duration
ANOVA	Group	F(1,21)=5.51, p=0.03	F(1,21)=8.9, p=0.01	F(1,19)=0.18, p=0.68	F(1,19)=0.15, p=0.7
	Delay	F(2.36,49.61+)=16.63, p<0.001	F(2.05,43.08+)=22.28, p<0.001	F(1.32,25.07+)=13.74, p<0.001	F(1.19,22.59+)=12.09, p=0.001
	Group*Delay	F(2.36,49.61+)=5.43, p=0.005	F(2.05,43.08+)=4.4, p=0.02	F(1.32,25.07+)=1.57, p=0.23	F(1.19,22.59+)=1.39, p=0.26

+ Greenhouse-Geisser correction

To investigate the influence of bone conduction and air conduction for auditory feedback, the pairwise linear correlation between the speech sound pressure level and the effect of DAF was calculated. The recorded peak sound pressure level was found to be correlated with the syllable number and the duration ratio at 200-ms delay for the voiced DAF experiment in the ASD group (syllable number: $r=0.67$, $p=0.03$; duration ratio: $r=0.72$, $p=0.02$).

Lombard effect experiment

When loud background noise was added to the auditory feedback, both the ASD group and the control group exhibited a significantly increased phonation duration (ASD group: $t(9)=7.16$, $p<0.001$) and sound pressure level (ASD group: $t(9)=5.38$, $p<0.001$).

Although both the ASD group and the control group exhibited a significant Lombard effect, the Lombard effect measured by phonation duration was significantly longer in the control group than in the ASD group ($t(21)=2.87$, $p=0.01$). Although the between-group difference in the Lombard effect measured by the sound pressure level was not significant ($t(21)=1.84$, $p=0.08$), it became significant ($t(19)=2.92$, $p=0.01$) after the removal of the outliers (one ASD participant and one NT participant whose Lombard effect measured by sound pressure level was larger than the cross-subject average Lombard effect measured by sound pressure level plus two standard deviations in each group).

Subjective report for delayed auditory feedback

Three two-way repeated measures ANOVAs, with factors Group and Delay, were conducted along with an evaluation of each description in the questionnaire (Table S2).

Table 3 The results of ANOVAs conducted for the results for the subjective report for self-agency perception of delayed auditory feedback

	Q1	Q2	Q3
Group	$F(1,21)=0.11$	$F(1,21)=0.1$	$F(1,21)=0.68$

	p=0.74	p=0.75	p=0.42
Delay	F(4,84)=4.72<br>P=0.002	F(4,84)=4.31<br>p=0.003	F(4,84)=4.76<br>P=0.002
Group*Delay	F(4,84)=1.34<br>p=0.26	F(4,84)=1.88<br>p=0.12	F(4,84)=0.37<br>p=0.83
